# Nasal septal lobular capillary haemangioma in West Africa sub-region: a case report

**DOI:** 10.4076/1757-1626-2-8952

**Published:** 2009-09-08

**Authors:** Ayotunde J Fasunla, Oluwatosin S Adebola, Clement A Okolo, Aderemi A Adeosun

**Affiliations:** 1 Department of Otorhinolaryngology, University College Hospital, PMB 5116, Ibadan, Nigeria; 2 Department of Pathology, University College Hospital, PMB 5116, Ibadan, Nigeria

## Abstract

**Introduction:**

Lobular capillary haemangioma is a rare vascular lesion of the nose. It is the aim of this communication to highlight the importance of considering this lesion as an important differential diagnosis of bleeding lesion in the nasal cavity.

**Case presentation:**

A case report of a 41-year-old female who presented with an obstructive, bleeding, pedunculated left nasal mass arising from the nasal septum in the anterior nasal cavity. An initial diagnosis of squamous papilloma was made and she had excision of the mass done under local anaesthesia. Tissue histology revealed lobular capillary haemangioma. The patient has been followed up for over 15 months and is still free of the lesion.

**Conclusion:**

To the best of our knowledge, this is the first report in the literature of lobular capillary haemangioma in the nasal septum in West Africa. The case is reported due to the rarity of this lesion in our environment.

## Introduction

Lobular capillary haemangioma is a benign vascular neoplasm which commonly affects the skin, mucosal of the oral cavity and tongue [1,2]. Nasal cavity mucosa involvement is very rare and if it occurs, the commonest affected site is the nasal septum [3,4]. Other sites that could be affected in the nasal cavity include nasal vestibule and middle turbinates [3,5]. Although the actual cause is unknown, factors such as microtrauma from nasal packing and prolonged intubation, and hormonal factors which include pregnancy had been implicated in the etiology of the lesion [3]-[8]. The most frequent symptoms of lobular capillary haemangioma include unilateral epistaxis and nasal obstruction by varied sizes of lobulated vascular masses with pedunculated stalk or wide base [3,5]. Complete excision of the lesion usually suffices in its successful treatment [3,6,7]. It is the purpose of this communication to report this first case of nasal septal lobular capillary haemangioma in an adult female in the West African sub-region; MEDLINE search has revealed no previous reports.

## Case presentation

The patient is a 41-year-old teacher, Yoruba by ethnic origin and Nigerian, who presented with a 3-week history of progressive, painless, left nasal growth associated with blood-stained left-sided nasal discharge and nasal blockage. This was preceded by nasal pricking. No excessive sneezing, nasal itch or fever. No pre-morbid history of nasal intubation, instrumentation or packing.

No other rhinologic, otologic or dental symptoms were present.

She had initial treatment at a secondary health institution where test aspiration of swelling was done two weeks prior to presentation with the disappearance of the swelling, the aspirate was bloody. However it recurred within a week thus necessitating the referral to our center.

There was no associated intercurrent medical condition or surgical operation. She does not snuff tobacco.

Clinical examination revealed a fit-looking middle-aged woman, not pale and afebrile.

**Figure 1:**
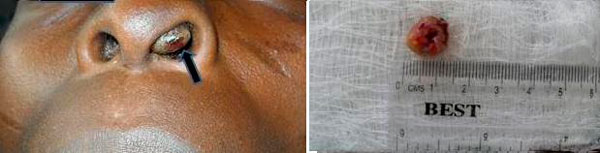
**Shows ulcerative, obstructive reddish brown polypoidal mass within the left nasal cavity (arrowed) and after excition**.

The nasal pyramid appeared normal. However, there was an obstructive left intranasal reddish brown, ulcerated polypoid mass which was attached to the anterior aspect of the tnasal septum by a pedicle. The mass was sensitive to touch and bled easily on contact (Figure 1).

Examination of the ears, oral cavity, oropharynx and the neck revealed essentially normal findings.

An initial assessment of left nasal septal mass most likely a squamous papilloma was made.

Her packed cell volume was 36% and her clotting profile was within normal limit. A plain paranasal sinus radiograph revealed a roundish area of lucency with sclerotic (opaque) rim in the left nasal cavity. The paranasal sinuses appeared clear and normal.

She had a per-nasal excision biopsy under local anaesthesia and haemostasis was secured with digital pressure application on the nasal pyramid.

**Figure 2:**
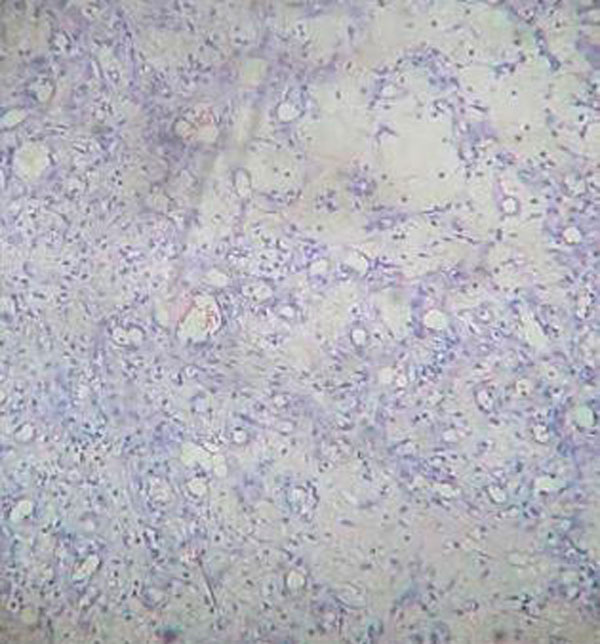
**Photomicrograph of lobular capillary haemangioma showing proliferation of small capillary channels in a loose fibromyxoid stroma which is infiltrated by chronic inflammatory cells (H & E ×400)**.

Histology report showed polypoidal fragments of tissues lined by stratified squamous epithelium with focal ulceration. The underlying stroma is composed of proliferating thin walled capillary channels arranged in lobular fashion. The stroma is infiltrated by acute and chronic inflammatory cells. This was conclusive of a Lobular Capillary Haemangioma (Figure 2).

She has been followed up for more than a year with complete healing of the site without any recurrence.

## Discussion

Nasal lobular capillary haemangioma, otherwise called pyogenic granuloma, is a rapidly growing benign vascular lesion which should now be considered in the differential diagnosis of a unilateral bleeding lesion in the nasal cavity.

The actual incidence of this condition in West Africa may not be known as it is a well known fact that hospital statistics in developing countries is a poor index of the true incidence of a disease condition. Many patients in both rural and urban areas of West Africa do not present to hospitals for sundry reasons ranging from cost of health care to distance from health facility. Increased awareness of this condition among healthcare practitioners is important in identifying the disease. It would also be necessary to send every excised lesion from the nasal cavity for histological diagnosis; this may lead to the identification of more cases of Lobular capillary hemangiomas and thereby show the true incidence of the disease.

Lobular capillary haemangioma is usually known to affect the skin and mucous layer of the oral cavity, while nasal involvement is rare [2]. The disease has been reported to affect more females than males [2,9]. Although it has been reported in all age groups, it is commoner in the third decade of life [2,9].

The clinical presentation of this lesion in the nasal cavity is similar to what other researchers have reported [2,4]-[8]. This patient presented with unilateral epistaxis and an obstructive reddish brown growth in the left nasal cavity. If one is not familiar with this lesion, it could easily be mistaken for other lesions such as angiofibroma, squamous papilloma etc.

A high index of suspicion is required in making the diagnosis. Although this lesion affects the anterior aspect of the nasal septum more commonly as seen in our patient, it has been reported in other endonasal sites which include the vestibule, middle turbinates and posterior part of the septum [3]-[5].

Review of literature has implicated micro-trauma from nasal packing and intubation as the most common predisposing factors [4]-[7]. Other etiological factors implicated include pregnancy and hormonal factors [8]. We suspect nasal trauma from habitual nasal pricking as the likely predisposing or etiological factor for the lesion in this patient. More work needs to be done to confirm nasal pricking as a predisposing factor for lobular capillary haemangiomas as such has not been reported in the literature.

The recurrence of this lesion is uncommon and no malignant transformation has been reported ([10]-[11]). There is no evidence of recurrence in this index case after 12 months of follow-up.

The surgical intervention carried out on the patient was that of a pernasal excisional biopsy under local anaesthesia which was sufficient for making histologic diagnosis as well as treating this patient as the mass was quite accessible on anterior rhinoscopy (Figure 1).

## Conclusion

Lobular capillary haemangioma is a rare neoplasm which should be considered in the differential diagnosis of rapidly enlarging vascular lesions within the nasal cavity. Pernasal excisional biopsy of the lesion under local anaesthesia is sufficient for making histologic diagnosis as well as treating this patient.

## Consent

Written informed consent was obtained from the patient for publication of this case report and accompanying images. A copy of the written consent is available for review by the Editor-in-Chief of this journal.

## Competing interests

The authors declare that there is no conflicting interest.

## Authors' contributions

AJF was the principal investigator and the conceiver of the idea for publication. He also performed the excision of the nasal mass. The literature search and the writing up of the manuscript were done by OSA and AJF. AAA is the owner of the patient and he revised the manuscript and supervised the patient's management. CAO prepared and reviewed the slide and the microfilm.
